# Developmental changes in human dopamine neurotransmission: cortical receptors and terminators

**DOI:** 10.1186/1471-2202-13-18

**Published:** 2012-02-15

**Authors:** Debora A Rothmond, Cynthia S Weickert, Maree J Webster

**Affiliations:** 1 Schizophrenia Research Institute, 405 Liverpool St, Darlinghurst NSW 2010 AU; 2 Neuroscience Research Australia, Schizophrenia Research Laboratory, Barker St, Randwick NSW 2031 AU; 3 University of New South Wales, Faculty of Medicine, School of Psychiatry, Hospital Rd, Randwick, NSW 2031 AU; 4 Stanley Laboratory of Brain Research, 9800 Medical Center Dr, Bldg. 2C-Rm C050, Rockville, MD, USA 20850

**Keywords:** DLPFC, postmortem, ADHD, schizophrenia, dopamine receptor, tyrosine hydroxylase, COMT, MAOA, MAOB, DRD

## Abstract

**Background:**

Dopamine is integral to cognition, learning and memory, and dysfunctions of the frontal cortical dopamine system have been implicated in several developmental neuropsychiatric disorders. The dorsolateral prefrontal cortex (DLPFC) is critical for working memory which does not fully mature until the third decade of life. Few studies have reported on the normal development of the dopamine system in human DLPFC during postnatal life. We assessed pre- and postsynaptic components of the dopamine system including tyrosine hydroxylase, the dopamine receptors (D1, D2 short and D2 long isoforms, D4, D5), catechol-*O*-methyltransferase, and monoamine oxidase (A and B) in the developing human DLPFC (6 weeks -50 years).

**Results:**

Gene expression was first analysed by microarray and then by quantitative real-time PCR. Protein expression was analysed by western blot. Protein levels for tyrosine hydroxylase peaked during the first year of life (p < 0.001) then gradually declined to adulthood. Similarly, mRNA levels of dopamine receptors D2S (p < 0.001) and D2L (p = 0.003) isoforms, monoamine oxidase A (p < 0.001) and catechol-*O*-methyltransferase (p = 0.024) were significantly higher in neonates and infants as was catechol-*O*-methyltransferase protein (32 kDa, p = 0.027). In contrast, dopamine D1 receptor mRNA correlated positively with age (p = 0.002) and dopamine D1 receptor protein expression increased throughout development (p < 0.001) with adults having the highest D1 protein levels (p ≤ 0.01). Monoamine oxidase B mRNA and protein (p < 0.001) levels also increased significantly throughout development. Interestingly, dopamine D5 receptor mRNA levels negatively correlated with age (r = -0.31, p = 0.018) in an expression profile opposite to that of the dopamine D1 receptor.

**Conclusions:**

We find distinct developmental changes in key components of the dopamine system in DLPFC over postnatal life. Those genes that are highly expressed during the first year of postnatal life may influence and orchestrate the early development of cortical neural circuitry while genes portraying a pattern of increasing expression with age may indicate a role in DLPFC maturation and attainment of adult levels of cognitive function.

## Background

The prefrontal cortex (PFC), particularly the dorsolateral prefrontal cortex (DLPFC), is markedly expanded and differentiated in the primate brain. In humans, the DLPFC [1,2] and hence adult level performance in working memory [3-5] may not fully mature until young adulthood. The functional integrity of the PFC is sensitive to modulation by catecholamines, particularly dopamine (DA) [6-8]. Indeed, DA is essential to the development and function of PFC controlled tasks of working memory, attention, behavioural flexibility, and planning that comprise executive function [9-12]. Cortical DA neurotransmission involves many genes and synchronised pre- and postsynaptic biochemical processes, interacting proteins, and enzymes. Several of these genes and proteins, including those involved in the synthesis of DA, in DA reception, and DA degradation were examined in this study to help define their roles during postnatal human brain development.

While previous studies have examined parameters of DA neurotransmission in the developing rodent, pig, and non-human primate PFC [13-18] few studies have done so in the human DLPFC over the postnatal lifespan. An early positron emission tomography (PET) report examined both dopamine receptor D1 (DRD1) and dopamine D2 receptor (DRD2) in 19-73 year olds and found an increase in the ratio of DRD1 to DRD2 with increasing age [19]. In a more recent study, in vivo PET was employed to evaluate DRD1 binding potential in twenty-eight late childhood/adolescent, and young adult healthy individuals and found DRD1 binding potential declined significantly with age in the DLPFC [20]. The few studies examining the developmental trajectories in human PFC of the DA degradation enzymes, monoamine oxidase A and B (MAOA, MAOB) [21] and catecholamine-*O*-methyltransferase (COMT) [22] showed developmental changes in protein but did not investigate mRNA expression. Previously, our laboratory measured developmental changes for tyrosine hydroxylase (TH) protein expression and DRD1, DRD2, and dopamine D4 receptor (DRD4) mRNA levels in cortical layers by *in situ *hybridization in the DLPFC. TH protein and DRD2 mRNA expression levels were high early in life and declined steadily with age whereas DRD1 mRNA expression was highest in young adulthood and DRD4 expression did not change significantly over the postnatal lifespan. Unfortunately, this study did not include any individuals between the ages of 1-13 years when numerous changes in brain function occur [23-25] and cognitive processes are developing, particularly in executive function [26,27].

In fact, it is during the school age period when disruptions in cortical DA neurotransmission are thought to contribute to the development of attention deficit/hyperactivity disorder (ADHD) [28]. However, it is likely that changes in DA neurotransmission in ADHD occur in the background of normal changes in cortical DA. Dysfunction of DA has also been implicated in another developmental neuropsychiatric illness striking in adolescence, schizophrenia [29,30], where working memory impairments are one of the hallmarks associated with this disorders [31-34]. Therefore, to better develop strategies to normalize DA signalling in an age-appropriate manner, more information is needed about how the DA system changes in human postnatal life. Utilizing microarray technology, changes occurring in the mRNA expression of dopamine-related gene products were examined across normal development of the human DLPFC and further confirmed by quantitative real-time PCR (qPCR). Because previous reports in the literature on the developmental patterns of DRD1 mRNA expression and binding are discrepant, we also included Western blot to determine the protein levels of DRD1 as well as to examine the protein levels of TH, MAOA, MAOB, and COMT. Our results indicate that the genes examined here are developmentally regulated over the protracted maturational period of the human DLPFC. The changes in gene and protein expression during postnatal life suggest that DA neurotransmission requirements of the DLPFC vary throughout development and it may be that genes like DRD2 and MAOA may have multiple functions that vary with age. Abundant DA synthetic capacity in the DLPFC appears to lessen as postnatal development progresses yet DA may take on a more targeted role in DLPFC functions such as in cognition, particularly after the first decade of life.

## Results

### Developmental Cohort and Demographic Variables

The seven age groups for this cohort were matched on the demographic variables of age, pH, PMI and RIN (summarised for each group in Table 1). Pearson correlations with the demographic variables and the genes of interest are detailed in Table 2. pH was found to positively correlate with DRD1 mRNA, DRD1 and MAOA proteins (all r > 0.24, p < 0.050) while PMI positively correlated with DRD2S, DRD5 mRNAs, and TH protein and was negatively correlated with DRD1, MAOA, and MAOB proteins (all r > 0.28, p < 0.050). All demographic variables correlating with genes of interest were entered as covariates in ANCOVAs examining age group effects.

**Table 1:** Developmental cohort demographic information

Age Group (#)	Age (Y)	pH	PMI	RIN	Gender
Neonate (11)	0.2 ± 0.04	6.50 ± 0.2	22.5 ± 5.1	7.8 ± 1.7	5F/6M
Infant (14)	0.5 ± 0.2	6.58 ± 0.2	16.9 ± 6.4	8.1 ± 1.0	5F/9M
Toddler (10)	2.8 ± 1.1	6.68 ± 0.3	21.2 ± 9.4	7.9 ± 1.1	5F/5M
School Age (9)	9.0 ± 2.7	6.63 ± 0.3	15.1 ± 4.7	7.9 ± 1.2	5F/4M
Adolescent (8)	16.9 ± 0.9	6.75 ± 0.1	15.5 ± 5.3	7.8 ± 0.9	2F/6M
Young Adult (9)	23.2 ± 1.8	6.67 ± 0.2	13.7 ± 8.3	8.6 ± 0.8	3F/6M
Adult (8)	43.4 ± 5.2	6.60 ± 0.3	13.4 ± 4.6	8.2 ± 0.6	3F/5M

**Table 2:** Pearsons product moment correlations for demographic variables and genes of interest

GENE		AGE		pH		PMI		RIN	
		**R**	**p**	**r**	**p**	**r**	**p**	**r**	**p**

**DRD1**	mRNA	0.28	0.039	0.108	0.439	0.33	0.014	0.047	0.737
	Protein	0.60	< 0.001	-0.28	0.021	0.33	0.006		
**DRD2S**	mRNA	-0.55	< 0.001	0.26	0.042	-0.13	0.317	-0.08	0.538
**DRD2L**	mRNA	-0.42	< 0.001	0.1	0.457	0.03	0.848	0.07	0.612
**DRD4**	mRNA	-0.30	0.057	0.02	0.898	-0.31	0.041	0.23	0.130
**DRD5**	mRNA	-0.33	0.012	0.26	0.049	-0.18	0.174	-0.07	0.622
**TH**	Protein	-0.57	< 0.001	0.37	0.002	-0.06	0.658		
**MAOA**	mRNA	-0.28	0.030	0.09	0.479	-0.01	0.925	-0.05	0.706
	Protein	0.76	< 0.001	-0.43	< 0.001	0.24	0.044		
**MAOB**	mRNA	0.49	< 0.001	-0.16	0.203	0.01	0.925	0.06	0.646
	Protein	0.5	< 0.001	-0.34	0.005	0.04	0.744		
**COMT**	mRNA	-0.11	0.386	0.09	0.475	-0.15	0.242	0.04	0.750
	Protein	-0.28	0.027	0.15	0.243	0.40	< 0.001		

### Tyrosine Hydroxylase

**Figure 1:**
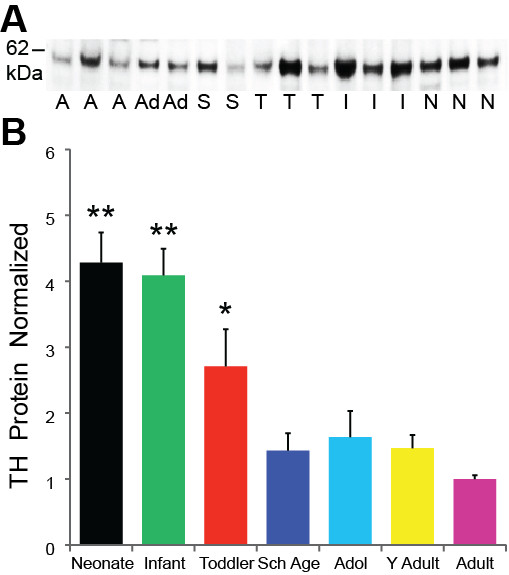
**(A) TH Western blot results demonstrating the expected 59 kDa band in representative cases from 6 of 7 age groups**. A = Adult, Ad = Adolescent, S = School Age, T = Toddler, I = Infant, N = Neonate. (B) TH protein expression is normalized to the adults. Post hoc analysis reveals the decline in TH with increasing age compared to neonate and infant groups as compared to all other age groups. ** = p < 0.01; * = p < 0.05.

A TH protein band was detected at the expected ~59 kDa size by Western Blot in each age group (Figure 1A). Western blots performed for the control protein β-actin, displayed no significant differences between the age groups (p = 0.53). TH protein levels varied significantly in intensity between the age groups (ANCOVA F = 8.95, df = 6,57, p < 0.001) and TH levels were highly and negatively correlated with age (regression r = -0.57, p < 0.001). Post hoc analysis showed that the neonate (p < 0.001), infant (p < 0.001), and toddler (p < 0.05) groups have significantly higher TH protein levels than the four older groups (Figure 1B). TH protein levels decrease markedly between the infant and school age periods and then remain fairly constant into adulthood. The largest change in expression occurs early in life with a 67% reduction in TH during the first 5 years after birth.

### Dopamine Receptors

Not all of the dopamine receptors were consistently detected by microarray in the DLPFC. DRD1 and DRD5 mRNA were expressed at detectable levels whereas DRD2 and DRD4 mRNA were not consistently expressed above 50% present (thus data not shown).

**Figure 2:**
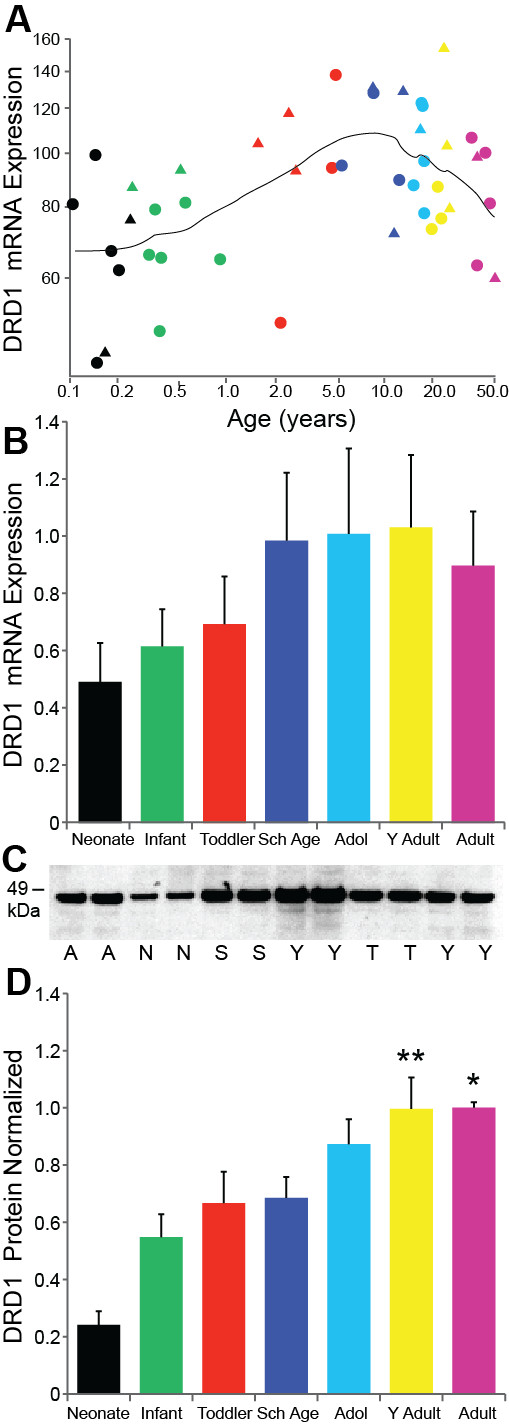
**(A) DRD1 microarray (p = 0.002) and (B) qPCR mRNA results show a similar pattern of increasing expression with age (p = 0.037) until adulthood**. (C) A representative Western blot for 6 of 7 age groups with the expected protein band at ~49 kDa. A = Adult, N = Neonate, S = School Age, Y = Young Adult, T = Toddler. (D) Protein expression increased robustly with age and appears to plateau during early adulthood. Adults and young adults had the highest levels of protein compared to neonates (both p < 0.001) infants (both p < 0.001), toddlers (p = 0.004, p = 0.006), and school age (p = 0.012, p = 0.009). Adolescents had greater levels of DRD1 protein than neonates (p < 0.001) and infants (p = 0.004). ** p ≤ 0.01, * p ≤ 0.05.

The microarray data revealed that opposite to TH protein, DRD1 mRNA increases significantly with age (regression r = 0.45, p = 0.002, ANOVA p = 0.014; Figure 2A), reaching peak expression in the school age period and then declining slightly after adolescence. The DRD1 qPCR mRNA data showed a similar pattern to that of the microarray data with increasing mRNA expression that weakly correlated with age (r = 0.28, p = 0.039) and there were no significant differences found in DRD1 mRNA by ANCOVA between the age groups (F = 1.17, df = 6,45, p = 0.337; Figure 2B).

DRD1 protein expression was strongly correlated with age (r = 0.60, p < 0.001). Western blot results show the expected ~ 49 kDa MW band for DRD1 protein at all time-points examined (Figure 2C). ANCOVA with pH and PMI revealed a significant effect of age group on DRD1 protein expression (F = 9.26, df = 6,58, p < 0.001). Post hoc analysis revealed that DRD1 protein levels rose substantially and steadily from neonatal to adult levels (Figure 2D). Adults and young adults had the highest levels of protein that significantly differed from neonates (both p < 0.001) infants (both p < 0.001), toddlers (p = 0.004, p = 0.006), and school age (p = 0.012, p = 0.009). The adolescent group also had greater levels of DRD1 protein compared to the neonates (p < 0.001) and infants (p = 0.004).

**Figure 3:**
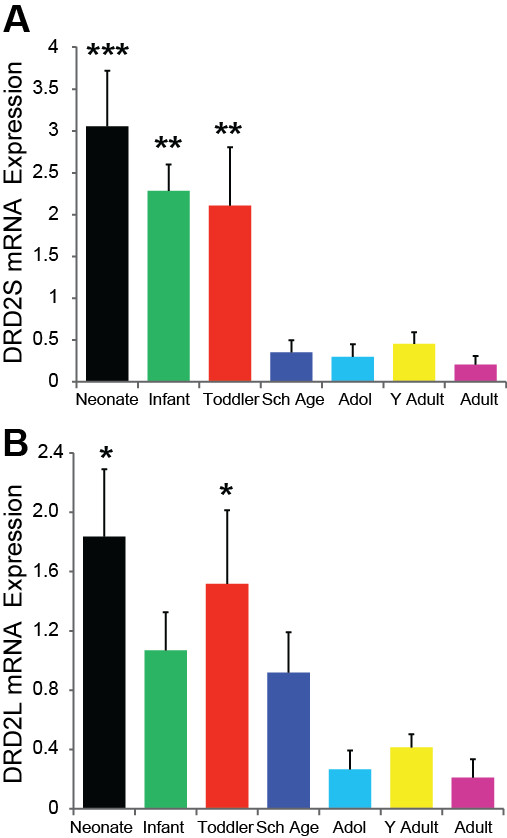
**(A) DRD2S (p < 0.001) and (B) DRD2L (p = 0.003) qPCR mRNA results**. (A) Neonates (p < 0.001), infant, and toddler groups (p < 0.01) had greater DRD2S mRNA than that of school age, adolescent, young adult & adult age groups. (B) DRDL mRNA followed a similar developmental pattern with the neonatal age group having the higher levels of DRD2L mRNA compared to school age (p = 0.050), adolescent (p < 0.001), young adult (p = 0.006), and adult age groups (p = 0.001). The toddler group had greater DRD2L mRNA than adolescents (p = 0.009), young adults (p = 0.041), and adults (p = 0.012). *** p ≤ 0.001, ** p ≤ 0.01, * p ≤ 0.05.

Using qPCR, the two DRD2 isoform mRNAs were easily and reliably detected. Unlike DRD1, DRD2S and DRD2L mRNA isoforms displayed a significant negative correlation with age (r = -0.55, p < 0.001; r = -0.42, p < 0.001 respectively). Both DRD2S (ANCOVA F = 7.55, df = 6,51, p < 0.001) and DRD2L (F = 3.64, df = 6,52, p = 0.004) mRNAs showed dramatic decreases in expression from the early age groups into adolescence. DRD2S mRNA expression was highest during the neonatal period with a substantial 84% downregulation of DRD2S mRNA between the toddler and school age years (Figure 3A). The neonate (p < 0.001), infant (p < 0.001), and toddler (p < 0.01) groups had significantly higher DRD2S mRNA from that of school age through adult groups. We detected significant differences between the neonate and toddler groups' DRD2L mRNA which both had higher levels compared to adolescent (p < 0.001; p = 0.009), young adult (p = 0.006; p = 0.041) and adult (p = 0.001; p = 0.012; Figure 3B) age groups.

**Figure 4:**
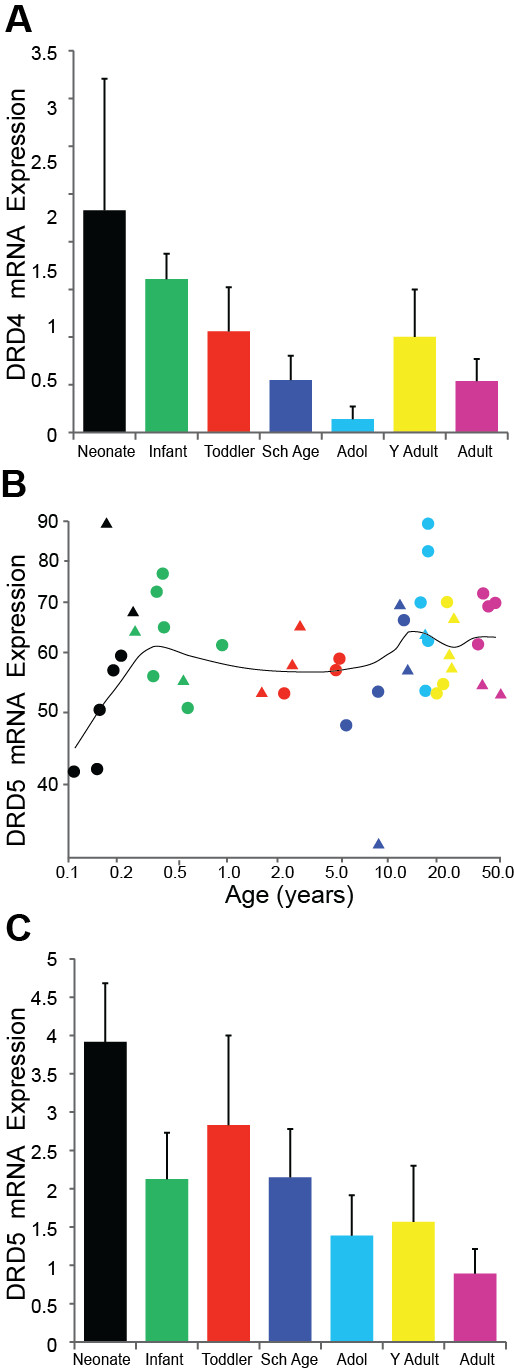
**(A) DRD4 qPCR mRNA expression in postnatal life (p = 0.147)**. (B) Results for the microarray DRD5 mRNA showed very little overall age related expression (regression p = 0.32). (C) DRD5 qPCR mRNA correlated negatively with age (p = 0.012) with no significant differences between the age groups (p = 0.353).

The DRD4 qPCR gene expression data was quite variable and was not significantly correlated with age (r = -0.30, p = 0.057) nor did we detect significant differences in DRD4 mRNA between the age groups (F = 1.71, df = 6,34, p = 0.147; Figure 4A).

The microarray results for DRD5 mRNA suggested that expression changed very little over the lifespan (regression r = 0.15, p = 0.324, ANOVA p = 0.277; Figure 4B). However, the DRD5 qPCR analysis showed a slight negative correlation with age (r = -0.33, p = 0.012) although we did not find statistically significant differences overall between the age groups (F = 1.14, df = 6,48, p = 0.354; Figure 4C).

### Dopamine Metabolism

Microarray analyses of mRNA encoding three enzymes involved in dopamine inactivation, MAOA, MAOB, and COMT were all found to significantly change expression levels during development.

### MAOA

**Figure 5:**
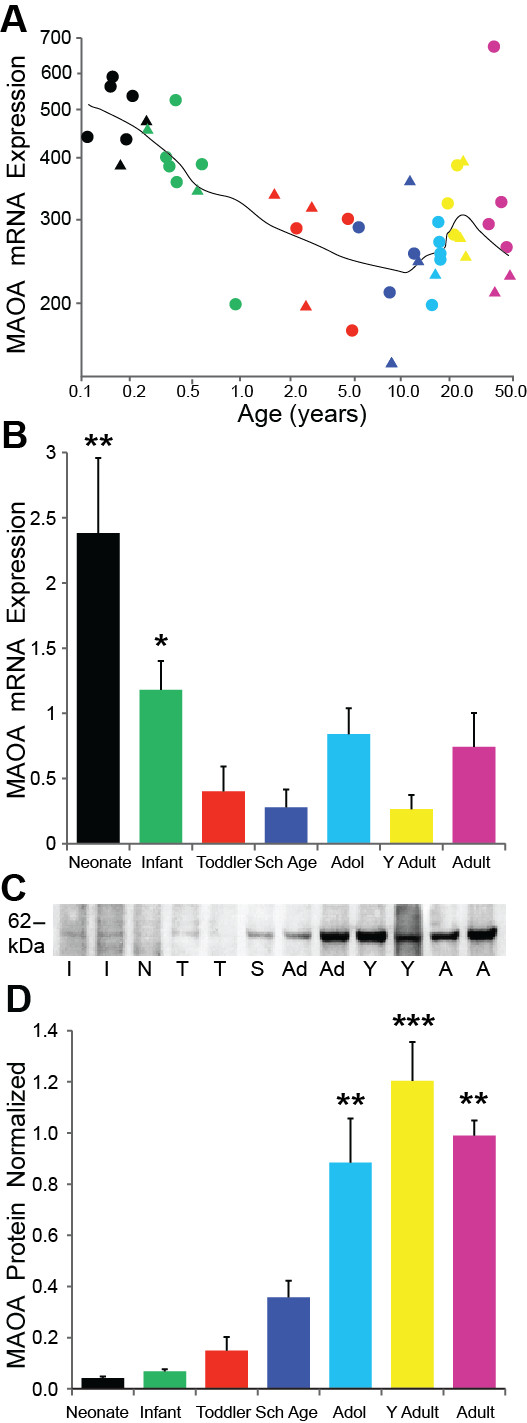
**(A) MAO-A microarray mRNA expression significantly changed with age (regression p < 0.001) with school age having the lowest mRNA levels; (B) Post hoc analysis of qPCR mRNA data confirmed that the school age group had the lowest expression (p < 0.001) and along with the young adult (p < 0.001) age group had reduced mRNA compared to the neonate group**. (C) A representative Western blot with the expected 61 kDa band for MAOA protein, and additional band at ~ 52 kDa. I = Infant, N = Neonate, T = Toddler, S = School Age, Ad = Adolescent, Y = Young Adult, A = Adult. (D) There is a dramatic difference in protein expression between age groups and opposite to that of MAOA mRNA. High immunoreactivity was observed in the older age groups, with young adults having significantly greater MAOA protein compared to neonates, infants, toddlers, school age groups (p < 0.001) as did the adolescents and adults (p < 0.01). *** p ≤ 0.001, ** p ≤ 0.01, * p ≤ 0.05.

Regression analysis of the microarray data revealed that MAOA mRNA was highly expressed in the neonate and infant groups and then decreased significantly with age (regression r = -0.56, p < 0.001, ANOVA p < 0.001; Figure 5A). Similarly, qPCR revealed a slight negative correlation of MAOA with age (r = -0.28, p = 0.030) and the mRNA changed significantly between the age groups (ANOVA F = 6.30, df = 6,55, p < 0.001; Figure 5B). Post hoc analysis showed that the neonates had significantly higher levels of mRNA compared to the infant (p = 0.003), toddler, school age (both p < 0.001), adolescent (p = 0.001), young adult and adult (both p < 0.001) age groups. Infants also significantly differed from the school age (p = 0.031) and young adult (p = 0.028) age groups.

Western blot confirmed MAOA protein expression at the ~61 kDa MW band for all age groups (Figure 5C). However, in stark contrast to the decrease with aging of MAOA mRNA, MAOA protein positively correlated with age (r = 0.76, p < 0.001) with significant differences found between the age groups (F = 27.08, df = 6,59, p < 0.001; Figure 5D). The highest level of MAOA protein was observed in the young adult group that significantly differed from neonates, infants, toddlers, school age (all p < 0.001) and the adolescent group (p = 0.010). In fact, MAOA protein levels more than doubled between the school age and adolescent age groups (p < 0.001). Adolescents and adults had greater MAOA protein compared to the neonatal, infant, toddler, and school age groups (p < 0.01).

### MAOB

**Figure 6:**
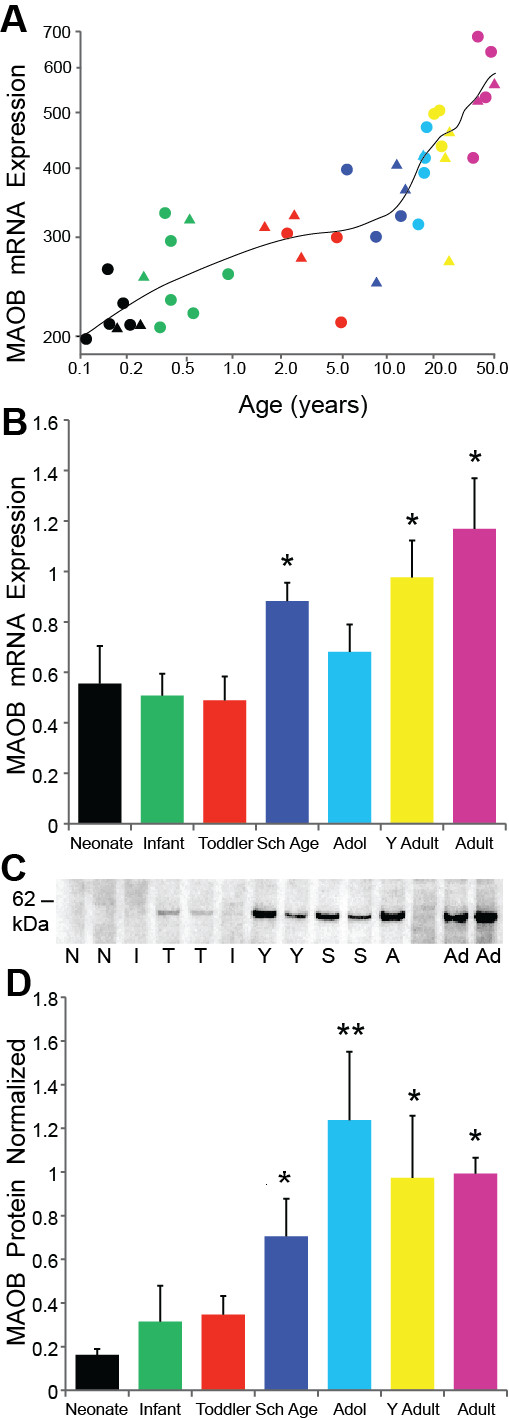
**(A) MAOB microarray mRNA data reveals the steep rise in expression with aging (regression p < 0.001); (B) qPCR mRNA results display similar pattern of up-regulation with age (p = 0.001)**. Post hoc analysis revealed the adult group possessed significantly higher mRNA compared to the neonate (p = 0.002), infant, toddler (both p < 0.001), and adolescent (p = 0.014) age groups. Young adults also had greater MAOB mRNA compared to the neonates (p = 0.023), infants (p = 0.007), and toddlers (p = 0.009) while the school age group had significantly higher mRNA than infants (p = 0.041) and toddlers (p = 0.045). (C) A representative Western blot indicating the identifying band of MAOB protein at 60 kDa in most age groups. N = Neonate, I = Infant, T = Toddler, Y = Young Adult, S = School Age, A = Adult, Ad = Adolescent. (D) MOAB protein expression peaks in adolescence (p < 0.001) and is much more abundant in both young adults and adults compared to the neonate (p < 0.001), infant (p < 0.01), and toddler (p ≤ 0.01) age groups. ** p ≤ 0.01, * p ≤ 0.05.

Opposite to the MAOA mRNA expression which decreased with age, microarray analysis revealed that MOAB mRNA increased dramatically with age peaking in adulthood (regression r = 0.83, p < 0.001, ANOVA p < 0.001; Figure 6A). The qPCR study confirmed that MAOB mRNA levels are positively correlated with age (r = 0.49, p < 0.001) and significantly changed between age groups (F = 4.22, df = 6,56, p < 0.001; Figure 6B). Post hoc analysis showed that adults had greater MAOB mRNA levels compared to neonatal (p = 0.002), infant, toddler (both p < 0.001), and adolescent (p = 0.014) age groups while the young adults also had higher levels of mRNA to that of neonates (p = 0.023), infants (p = 0.007), and toddlers (p = 0.009) as did the school age group compared to infants (p = 0.041) and toddlers (p = 0.045).

MAOB immunoreactivity with the expected ~60 kDa MW band was present at all ages (Figure 6C). As with the mRNA, the MAOB protein levels increased significantly with age (r = 0.50, p < 0.001) and differed between the age groups (ANCOVA F = 4.79, df = 6,60, p < 0.001) with peak MAOB protein expression in the adolescent group. Post hoc analysis showed that the adolescent group had significantly higher levels than the neonate, infant, toddler (all p < 0.001) and school age (p = 0.031) age groups. The neonates had the lowest protein levels of all age groups with significant differences compared to the school age (p = 0.018), adolescent, young adult and adult (all p < 0.001) age groups (Figure 6D).

### COMT

**Figure 7:**
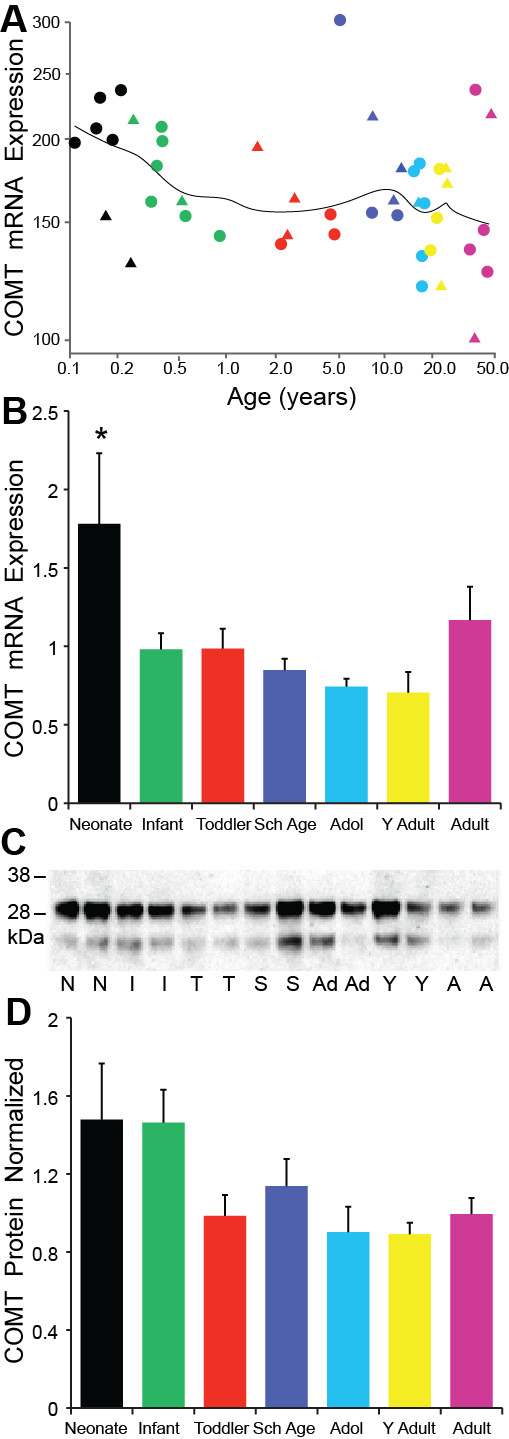
**(A) COMT microarray (regression p = 0.025) and (B) qPCR mRNA results (p = 0.018) reveal a subtle pattern of early high COMT expression**. The neonatal group had mRNA levels 35-60% higher than all other age groups (p ≤ 0.001-p ≤ 0.05). (C) A representative Western blot gel depicts two bands at ~30 kDa & ~24 kDa of COMT protein that is consistent with both membrane bound (32 kDa) and the soluble form (24 kDa); N = Neonate, I = Infant, T = Toddler, S = School Age, Ad = Adolescent, Y = Young Adult, A = Adult. (D) The COMT protein results follow a pattern similar to the mRNA data with higher expression early in life that gradually declines over time but was not statistically significant. * p ≤ 0.05.

The microarray data showed COMT mRNA decreased throughout development with slightly higher levels of expression at birth that gradually declined with age (regression r = -0.33, p = 0.024, ANOVA p = 0.252; Figure 7A). The qPCR mRNA study also showed a significant decrease in COMT expression between the age groups (ANOVA F = 2.83, df = 6,58, p = 0.018; Figure 7B) but did not correlate with age (r = -0.11, p = 0.386). Neonates had significantly greater COMT mRNA than the infant (p = 0.006), toddler (p = 0.013), school age (p = 0.005), adolescent (p = 0.002), young adult (p = 0.001) age groups.

Western blot for COMT showed two major bands of protein with the ~30 kDa membrane bound protein forming the most robust band and another band forming at ~24 kDa consistent with the soluble protein form. Both bands were present in all age groups (Figure 7C). Age showed a weak negative correlation with expression for the ~30 kDa COMT protein (r = -0.28, p = 0.027) but not with the ~24 kDa protein (r = -0.15, p = 0.238). There were no significant differences for age group with either COMT protein band (p > 0.05; Figure 7D).

## Discussion

Various components of the dopamine system displayed distinct and robust changes in both transcript and protein levels during postnatal development. Thus, the prefrontal cortical DA system shows quite dynamic developmental profiles in relation to DA synthesis, receptor signalling, and DA breakdown which all change as humans grow and mature. Many DA receptor mRNAs showed dynamic changes across development. The most commonly observed developmental pattern was to have the highest expression in the youngest age groups, within the first five years of life followed by a decline in expression with age. The expression of DRD2S, DRD2L, and DRD5 mRNAs all displayed this pattern of downregulation with maturation as did TH and MAOA mRNA. MAOA was the only molecule that showed a pronounced decoupling between mRNA and protein expression with the protein showing an increase with age. MOAB and DRD1 (mRNA and protein) showed a gradual increase in expression with age. Interestingly, DRD1 was unique among the dopamine receptors in terms of its developmental profile and our results suggest that there is an increased role for DRD1 as the human cortex matures.

Previous reports suggest that by early adulthood DRD1 is the most prevalent receptor in the PFC followed by DRD2, DRD4, and DRD5 expression [18,35]. The results reported here show that while all other receptors are decreasing with age, DRD1 mRNA and protein levels are increasing in postnatal life. The increase in DRD1 mRNA expression with age is consistent with our earlier report in a different cohort that showed an increase in DRD1 mRNA expression in multiple cortical layers in the DLPFC during development [36] with similar peaks of expression during adolescence and young adulthood. As more age groups are represented in this study our results here suggest that a substantial increase in DRD1 mRNA is evident even earlier at around the time of school age years where mRNA levels were very similar to that of adolescent and young adult age groups.

The DRD1 protein peak in expression appears to occur a few years later in life than that of mRNA expression. DRD1 is critical to PFC cognitive functioning and in particular working memory [37] and these cognitive processes may not fully mature until the third decade of life [38,39] Thus, the change in DRD1 from periadolescence into young adulthood happens during a time in development when the cortex and our cognitive behaviour are also maturing suggesting that DA-DRD1 may have an integrative role in higher cortical function.

While DRD1 mRNA and protein expression is low early in cortical development, DRD5 expression is at its highest expression. This pattern of DRD5 expression which is opposite to the DRD1 pattern is interesting, given that both receptors are almost indistinguishable pharmacologically [40]. While all of the DA receptors have been shown to play some role in cognition [41,42], DRD5 is consistently co-localised with DRD1 on pyramidal neurons in the PFC [43]. DRD5 has a ten-fold higher affinity for DA as compared to DRD1 [40] and our finding of increased early expression of DRD5 suggests that this receptor may play a more salient role in early postnatal cortical development than DRD1. Thus, it may be that each receptor provides a differential contribution to DA's influence over pyramidal neurons, dependent upon whether it is in early life or at maturation [44].

To date, there are no published reports of the DRD2 short and long isoforms in developing primate or human PFC and results in the developing rodent cerebral cortex have been inconsistent. A recent postnatal study in rodent cortex showed DRD2S and DRD2L mRNA expression peaking in early development which is similar to our results [45]. In contrast, Mack et al., (1991) reported that in whole rat brain extracted mRNA, both isoforms increase expression throughout pre- and postnatal development with the highest levels occurring in adulthood [46]. A third study examined the isoforms in whole brain and found DRD2S peaking at 14 days (approximately infant age in humans) and DRD2L at 28 days (approximately school age years) with mRNA levels declining thereafter [47]. We find that in human PFC, DRD2 transcripts exhibit high expression at birth that declines with age. Our studies show that the developmental decrease in DRD2S (before school age) may occur prior to DRD2L (after school age) suggesting that during the school age period in normal children the balance of DRD2 isoform signalling may favour the long isoform of the receptor. Our earlier study examining DRD2 mRNA in PFC showed that the mRNA was greatest during the first few months of postnatal life in all cortical layers. Increased DRD2 mRNA early in development that is localised to both excitatory [48,49] and inhibitory [50,51] neurons, may enable DA to modulate neuronal cell types, especially immature GABA neurons because interneurons, which show protracted maturation, [52] are still differentiating in human DLFC postnatally [53]. Also there is still considerable growth of cell soma, dendrites, and synapses occurring within the first five years of life [54] when DRD2 and DRD5 mRNA levels are high and DRD1 levels are lowest. Since DRD2 is negatively coupled to adenylate cyclase, high DRD2 levels may be important for attenuating pyramidal neuron activity when DRD5 is high early in life.

Rodent studies have shown varied results in the developmental pattern of DRD4 mRNA expression [18,47,55] as have human studies [56,57]. The results presented here suggest that DRD4 mRNA expression may be variable throughout postnatal life. Our previous postnatal study of laminar patterns of DRD4 expression did not include the toddler or school age groups but did report that the highest levels of DRD4 mRNA expression occurred in the infant group, particularly in layer V and VI [36]. The lack of robust change in DRD4 we find here suggests that age may not be a strong regulator of DRD4 or that maintaining fairly steady DRD4 is important throughout postnatal life.

As anticipated, TH protein is in greater abundance during the first decade of life and recedes with age [36]. It may be that TH and hence DA are necessary in greater abundance during prenatal and early postnatal brain development in order to stimulate the establishment of other neurons and connections. Indeed, TH fibres appear early in embryonic cortex in the telencephalic wall as early as gestational week 8 in humans [58]. The crucial role of TH in overall development is reinforced by the fact that TH knockout mice do not survive beyond E15.5 [59]. As the PFC develops postnatally, the amount of TH and DA synthesis required may be much less. Our results of high levels of TH early in life in the human DLPFC, now found in two distinct cohorts [36], suggests that there is a large synthetic demand for dopamine early in life when cortical pyramidal neurons (dendritic and appositions) are still maturing [15] and inhibitory interneurons are still migrating and differentiating [53]. It is also possible that while TH and thus DA synthesis decline, DA innervation becomes more targeted and circuitry refined so that less DA is required to be effective [15].

Another way to change the parameters of DA action is to change the time course of action. The genes responsible for the inactivation via degradation of DA were found to vary widely in their developmental profiles, with very significant and distinct patterns of expression and in some cases completely opposite profiles from transcription to protein. MAOA mRNA and COMT mRNA and protein exhibited the highest levels of expression in the earliest age groups, suggesting that DA synthesis may be highest early in life and that increased metabolism of DA (via MAOA) may be required to maintain a biochemical balance. But MAOA protein, MAOB mRNA and protein levels increased with age suggesting that later in life there may be increased breakdown of DA through MAOs and that DA's action may be temporarily restricted.

It is not clear why MAOA mRNA expression declined across development whereas MAOA protein increased throughout development to reach peak levels in young adulthood. Our findings do not appear spurious given that the MAOA mRNA data was confirmed in both the microarray and qPCR studies and the Western results show the immunoreactive band of the expected size and to be robustly expressed. Thus the MAOA mRNA may not be efficiently translated into protein or the MAOA protein may be very unstable early in life. It is also possible that there is a presynaptic increase in MAOA protein due to mRNA that is synthesized in the brain stem and the protein is transported to axon terminals such that mRNA and protein levels would appear uncoupled. Previous binding studies of MAOA in rodent suggest a profile similar to our protein results where MAOA expression increases from P0, plateau around P21 and gradually declines through adulthood [60-62]. In addition, mRNA studies in the rodent are consistent with our mRNA results where MAOA expression declines [63]. However, the low level of MAOA protein in early postnatal life is in contrast to published data on radiolabeled MAOA activity in postmortem human frontal cortex that showed the highest MAOA activity in humans less than one year of age [21]. Further study is needed to resolve if species differences exist or if discrepancies in the developmental profiles of MAOA mRNA, protein expression, and activity levels exist.

Unlike MAOA, MAOB mRNA and protein both increase throughout postnatal life. Having low levels of MAOA protein and MAOB protein expression early in life while DA synthesis is presumably highest and DA breakdown slower due to lower levels of MAOA protein and MAOB protein with perhaps only COMT at higher levels, suggests that the overall actions of dopamine may be prolonged in the infant PFC where presumably more DA is available to be released and where there may be a delay in degradation through MAOA and MAOB. This is particularly true for MAOB which has been shown to have a higher affinity for DA than MAOA [64] and both MOAs have affinities for other catecholamines as well as serotonin and this may have a more broad influence over monoamine developmental availabilities. Hence, MAOB may play an increasingly significant role in cortical DA metabolism at maturation and in adult life.

COMT mRNA is more prominent in the PFC than in subcortical brain regions [65]. Although the noradrenaline transporter [66,67], vesicular monoamine transporter 2 (VMAT2) and MAO [64,68] contribute to DA elimination, COMT is responsible for approximately ~ 60% of all DA degradation in the PFC [69] and our results suggest that this may be higher earlier in postnatal life. The role of COMT in maintaining DA neurotransmission is clearly an important one. Indeed, the TH data would indicate that DA synthesis and presumably DA itself, is extremely abundant early in life with slightly higher levels of COMT protein. COMT mRNA and protein displayed a decrease in expression. The higher gene expression of COMT early in postnatal development is consistent with a previous report in rat where COMT mRNA was highly visible in cortex, hippocampus, and striatum at day P1 and with higher mRNA levels at P1 in hypothalamic nuclei that diminished with age [70]. However, it is not likely that COMT mRNA and protein levels alone predict COMT activity. In a previous study in human DLPFC we found increasing COMT enzymatic activity with age was associated with the COMT Val^158^Met polymorphism [22], although genotype effects on mRNA levels [71,72] and protein [22,73] levels are not consistent. Further studies are needed to understand the developmental relationship between COMT genotype, COMT synthesis, COMT activity, and DA levels.

## Conclusions

Taken together these results indicate that pre- and postsynaptic genes of the dopamine system studied here are developmentally regulated over the protracted maturational period of the human DLPFC. The changes in gene and protein expression during postnatal life suggest that DA neurotransmission requirements of the DLPFC vary throughout development and it may be that genes like DRD2 and MAOA may have multiple functions that vary with age. Several genes that showed high early gene expression had their lowest levels of expression around the school age and adolescent periods (DRD2S, DRD2L, and MAOA). Genes that had increased expression with age may be associated with healthy adult cognition and behaviour. Abundant DA in the DLPFC appears to lessen as postnatal development progresses yet DA may take on a more targeted role in DLPFC functions such as in cognition, particularly after the first decade of life. Our results underscore the need for greater understanding of how the drugs used to treat ADHD, which increase cortical DA neurotransmission and are typically given in school age children [74], and schizophrenia, which block DA neurotransmission (DRD2) and are often given in adolescence, [75] may alter these normal developmental trajectories.

## Methods

### Subjects

Postmortem samples from DLPFC (Brodmann's area 46) from 69 cases ranging in age from 6 weeks to 49 years were obtained from the National Institute of Child Health and Development Brain and Tissue Bank for Developmental Disorders (NICHD Contract NO1-HD8-3283; IRB approval H-20765) [76]. Briefly, the cases included 41 males, 28 females, 37 African Americans, 30 Caucasians, and 2 Hispanics, and were grouped as neonates (0.1-0.24 years), infants (0.25 - 0.9 years), toddlers (1-5 years), school-age (6-12 years), adolescents (14-17 years), young adults (20-25 years) and adults (35 -50 years). The age groups were matched for mean postmortem interval (PMI) and pH. All subjects were free of neurological and psychiatric symptoms at the time of death.

### RNA Extraction, Purification, and Quality Assessment

In RNase-free conditions, 0.5 g of grey matter was excised from a frozen 3 mm coronal DLPFC tissue slice of each case then pulverized and weighed while frozen, and stored at -80°C. Total RNA was extracted from ~ 300 mg of DLPFC using a modified TRIzol^® ^Reagent extraction procedure as previously described [77]. RNA integrity number (RIN) measurements were calculated for each sample and are listed in Table 1 using the Agilent 2100 Bioanalyzer system. Cases were excluded from the qPCR and microarray studies with a RIN value below 6.0. Samples used for the microarray study were further purified through a Qiagen RNeasy Mini kit (Cat # 74104, Qiagen Inc.) according to the manufacturer's protocol. For the DRD1 and DRD5 qPCR experiments, an aliquot of each total RNA sample underwent DNase treatment using DNA-*free *(Cat # AM1906, Ambion, Life Technologies, Inc.) to remove any genomic DNA contamination.

### Quantitative real-time PCR (qPCR)

3 μg of RNA from each sample was converted into cDNA using the First-Strand SuperScript II^® ^synthesis system for qPCR (Cat # 11904-018, Invitrogen, Life Technologies, Inc.) following the manufacturer's protocol. qPCR reactions were performed on an ABI Prism 7900 Fast sequence detection system in a 384-well format (Applied Biosystems, Life Technologies, Inc.) using the TaqMan gene expression system (part numbers for each assay are listed in Table 3). Housekeeping genes that did not change expression with development were used for the geomean calculations: beta-2-microglobulin (B2M), hydroxymethylbilane synthase (HMBS), glucuronidase, beta (GUSB), and peptidylprolyl isomerase A (PPIA). The housekeeping genes and the subsequent geomeans were tested by Pearsons product moment correlations and did not significantly correlate to any age group (all r < 0.13, p > 0.27). All samples were run in triplicate and measured in the same plate for each gene of interest. Triplicate outliers were determined by Grubbs Test (GraphPad), removed, and the mean recalculated. NTC wells resulted in no detectable signal. The change in mRNA expression was calculated using the average measurement of the adult cases as the calibrator for the target genes and normalized to the geometric mean of the housekeeping genes for each experiment using the ΔΔCt method [78].

**Table 3:** Applied Biosystems TaqMan gene expression assay part numbers

Gene Name (Gene Symbol)	Taqman Assay
Glucuronidase, beta (GUSB)	Hs99999908_m1
Peptidylprolyl isomerase A (Cyclophilin A-PPIA)	Hs99999904_m1
Beta-2-microglobulin (B2M)	Hs99999907_m1
Hydroxymethylbilane synthase (HMBS)	Hs00609297_m1
Catechol-O-methyltransferase (COMT)	Hs00241349_m1
Dopamine receptor D1 (DRD1)	Hs00265245_s1
Dopamine receptor D2 long isoform (DRD2L)	Hs01024460_m1
Dopamine receptor D2 short isoform (DRD2S)	Hs01014210_m1
Dopamine receptor D4 (DRD4)	Hs00609526_m1
Dopamine receptor D5 (DRD5)	Hs00361234_s1
Monoxidase A (MAOA)	Hs00165140_m1
Monoxidase B (MAOB)	Hs011006243_m1

### Microarray RNA Study

The microarray study contained six-eight cases in each of seven age groups totalling forty-eight cases. RNA was processed through the Affymetrix preparation protocol [http://www.affymetrix.com, [79]] hybridized to HG-U133 version 2.0+ (GeneChips, Affymetrix) as previously described [80]. Affymetrix Microarray Suite (MAS 5.0) was used for image processing and data acquisition. The Bioconductor package was used to compute normalized expression values from the Affymetrix '.cel' files.

### Protein Extraction and Quantitative Determination

Protein was extracted from approximately ~ 100 mg of DLPFC, thawed on wet ice, and homogenized with Tris-glycerol extraction buffer in the presence of protease inhibitors at the ratio of 10 mL of buffer to 1 g of tissue as previously described [81]. Aliquots (50 μl) of the samples were placed into individual microcentrifuge tubes and stored at -80°C.

To quantitate protein expression, 1-10.6 μg (Table 4) of total protein determined by Bradford assay from each case was assessed by Western blot. Samples were run on 10% polyacrylamide gels at 120 V for 2 hr and blotted onto a nitrocellulose membrane for 1.5 hr at 30 V. Primary antibody dilutions (Table 4) were prepared in 3% normal goat (Vector Labs) and 2% non-fat dry milk in TBS-T and then incubated for 16-72 hr on an orbital shaker at 4°C. Following washes in TBS-T, secondary antibody dilutions were prepared in 4% normal goat or donkey serum in TBS-T and applied for 2 hr at RT. The gels were rinsed, exposed to ECL plus chemiluminescent substrate detection reagents (Amersham/GE Healthcare) for the visual detection of the immunopositive bands and finally exposed to Kodak Bio-Max MR film. Values measured from each sample were normalized to the adults (100%) run on the same gel.

**Table 4:** Primary (1^o^) and secondary (2^o^) antibody (Ab) part numbers, dilutions, and incubation information

**1**^o ^**Antibody** (Conc)	Company (Part No)	**2**^o ^**Antibody** (Conc)	Company (Part No)	Protein (μg)	Incubation Time (hrs)
COMT Rabbit IgG (1:3000)	Millipore (AB 5873)	Goat anti-rabbit (1:10000)	Millipore (AP307P)	1.0	24
DRD1 Rabbit IgG (1:1000)	ABCAM (40653)	Goat anti-rabbit (1:100000)	Millipore (AP307P)	8.5	18
MAOA (C19) Goat IgG (1:1000)	Santa Cruz (sc-18396)	Donkey anti-goat (1:100000)	Santa Cruz (sc-2020)	6.0	18
MAOB (D16) Rabbit IgG (1:3000)	Santa Cruz (sc-18402)	Donkey anti-goat (1:100000)	Santa Cruz (sc-2020)	3.0	18
TH Mouse IgG (1:1000)	Millipore (MAB 318)	Goat anti-mouse (1:10000)	Millipore (AP124)	10.6	72

### Statistical Analyses

Regression and Analysis of Variance (ANOVA) statistical analyses for the Affymetrix microarray data was performed using R and Bioconductor software. Genes that did not reach criteria for 50% present or that had expression levels below the limit of detection (50 units) were not reported in microarray results. Statistical analyses for qPCR and Western blot data were conducted using Statistica (StatSoft Inc., 2005, Statistica ver 7.1). Pearsons product moment correlation analyses were conducted for the impact of demographic variables (age, pH, PMI, RIN) on the genes of interest. ANOVAs were performed to assess age group differences for each gene of interest. ANCOVAs were performed where significant correlations with demographic variables were detected. Fisher LSD post hoc tests were used to determine significant differences between the group means. Prior to qPCR statistical analyses the normalized dataset was assessed for population outliers within each developmental group (Grubbs Test, GraphPad). Significance was set at p ≤ 0.050.

## List of abbreviations

ADHD: Attention deficit hyperactivity disorder; AD: Adolescents; A: Adults; B2M: Beta-2-microglobulin; COMT: Catechol-*O*-methyltransferase; DA: Dopamine; DLPFC: Dorsolateral prefrontal cortex; DRD1: Dopamine D1 receptor; DRD2S: Dopamine D2 receptor short isoform; DRD2L: Dopamine D2 receptor long isoform; DRD4: Dopamine D4 receptor; DRD5: Dopamine D5 receptor; GUSB: Glucuronidase; beta; HMBS: Hydroxymethylbilane synthase; I: Infants; mRNA: Messenger RNA; MAOA: Monoamine oxidase A; MAOB: Monoamine oxidase B; PMI: Postmortem interval; PPIA: Peptidylprolyl isomerase A; qPCR: Quantitative real-time PCR; RIN: RNA integrity number; S: School age; T: Toddler; TH: Tyrosine hydroxylase; Y: Young adult.

## Competing interests

The authors declare that they have no competing interests.

## Authors' contributions

DAR carried out the cDNA and qPCR experimental procedures, performed the statistical analyses and interpretation of the qPCR and Western blot data, prepared all figures and tables, and assisted with the writing of the manuscript. CSW contributed to the conception and design of the microarray, qPCR and protein studies, tissue acquisition, interpretation of the data, and assisted with critical revisions of the manuscript. MJW contributed to the conception and design of the microarray, qPCR, and protein studies, tissue acquisition and preparation of RNA, interpretation of data, and assisted with the writing of the manuscript. All authors read and approved the final manuscript.
